# Beneficial Roles of Cellulose Patch-Mediated Cell Therapy in Myocardial Infarction: A Preclinical Study

**DOI:** 10.3390/cells10020424

**Published:** 2021-02-17

**Authors:** Rossana B. Simeoni, Bassam F. Mogharbel, Julio C. Francisco, Nelson I. Miyague, Ana C. Irioda, Carolina M. C. O. Souza, Daiany Souza, Priscila E. Ferreira Stricker, Nádia Nascimento da Rosa, Clayton F. Souza, Celia R. Cavichiolo Franco, Maria-Rita Sierakowski, Eltyeb Abdelwaid, Luiz C. Guarita-Souza, Katherine A.T. Carvalho

**Affiliations:** 1 Experimental Laboratory of Institute of Biological and Health Sciences of Pontifical Catholic University of Paraná (PUCPR), Street Imaculada Conceição, 1155, 80215-901 Curitiba, Paraná, Brazil; rossanabaggio@gmail.com (R.B.S.); julio.apfr@gmail.com (J.C.F.); nelson.miyague@gmail.com (N.I.M.); guaritasouzalc@hotmail.com (L.C.G.-S.); 2 Cell Therapy and Biotechnology in Regenerative Medicine Research Group, Pelé Pequeno Príncipe Research Institute & Pequeno Príncipe Faculties, Ave., Silva Jardim, 1632, 80240-020 Curitiba, Paraná, Brazil; bassamfm@gmail.com (B.F.M.); anairioda@gmail.com (A.C.I.); carolinamcos@yahoo.com.br (C.M.C.O.S.); daianys.bio@gmail.com (D.S.); priscilaeferreira@gmail.com (P.E.F.S.); nadianr@gmail.com (N.N.d.R.); 3 Biopol, Chemistry Department, Federal University of Paraná, Avenue Cel. Francisco Heráclito dos Santos, 200, 81530-900 Curitiba, Paraná, Brazil; clayton.biopol@gmail.com (C.F.S.); mariarita.sierakowski@gmail.com (M.-R.S.); 4 Chemistry Undergraduate Program, School of Education and Humanities of Pontifical Catholic University of Paraná (PUCPR), Street Imaculada Conceição, 1155, 80215-901 Curitiba, Paraná, Brazil; 5 Molecular Biology Department, Federal University of Paraná, Avenue Cel. Francisco Heráclito dos Santos, 100, 81530-900 Curitiba, Paraná, Brazil; crcfranc@terra.com.br; 6 Feinberg School of Medicine, Feinberg Cardiovascular Research Institute, Northwestern University, 303 E. Chicago Ave., Tarry 14–725, Chicago, IL 60611, USA; eltyeb.abdelwahid@northwestern.edu

**Keywords:** implant, bacterial cellulose patch, myocardial infarction, cell therapy, delivery

## Abstract

Biological scaffolds have become an attractive approach for repairing the infarcted myocardium and have been shown to facilitate constructive remodeling in injured tissues. This study aimed to investigate the possible utilization of bacterial cellulose (BC) membrane patches containing cocultured cells to limit myocardial postinfarction pathology. Myocardial infarction (MI) was induced by ligating the left anterior descending coronary artery in 45 Wistar rats, and patches with or without cells were attached to the hearts. After one week, the animals underwent echocardiography to assess for ejection fraction and left ventricular end-diastolic and end-systolic volumes. Following patch formation, the cocultured cells retained viability of >90% over 14 days in culture. The patch was applied to the myocardial surface of the infarcted area after staying 14 days in culture. Interestingly, the BC membrane without cellular treatment showed higher preservation of cardiac dimensions; however, we did not observe improvement in the left ventricular ejection fraction of this group compared to coculture-treated membranes. Our results demonstrated an important role for BC in supporting cells known to produce cardioprotective soluble factors and may thus provide effective future therapeutic outcomes for patients suffering from ischemic heart disease.

## 1. Introduction

Myocardial infarction and heart failure are major causes of death in developed countries [1]. Several deleterious effects are known to occur in both the infarcted and non-infarcted myocardial tissues, including cardiomyocyte loss, cardiomyocyte lengthening, left ventricular wall thinning, infarct expansion, cardiomyocyte hypertrophy, and collagen accumulation [2,3,4].

Molecular studies have revealed that regeneration of tissues in various systems can be augmented by growth factors such as platelet-derived growth factor (PDGF), bone morphogenetic proteins (BMPs), and transforming growth factor-β (TGF-β) [5]. Nevertheless, the expensive cost and rapid growth factor degradation remain as main obstacles. Many types of materials are now under extensive investigations to find promising scaffolds for tissue engineering. The ideal scaffold should meet specific criteria, being biocompatible, matching surrounding tissues, providing chemical stability or degradability, affording mechanical strength, and allowing cell adhesion and proliferation. In natural tissues, the extracellular matrix composition, cell density, and physiological properties are often nonhomogeneous. A great challenge of tissue engineering is the distribution of cells throughout tissue engineering scaffolds. These biomimetic materials should resemble the natural tissue and extracellular matrix (ECM) to provide the targeted area [6]. Mimicking natural conditions in both the tissue and ECM requires proper adhesion and growth properties that maintain normal tissue structure [7]. Biopolymers involving cellulose were tried previously for developing scaffolds and revealed successful outcomes [8,9,10,11,12].

At the molecular level, bacterial cellulose (BC)-based aqueous gel-like biomaterial has been used to quantify transcriptomics and proteomics in cell culture. Interestingly, statistical analysis of 12,475 transcripts and 7831 proteins documented significant differences, indicating its role in major gene-dependent functional responsiveness [13]. Cellulose is a linear homopolysaccharide that consists of glucose (d-glucopyranose) linked by glycosidic β (1-4) linkages. Its polymerization degree varies widely from 2400 in some plants, such as the horsetail (*Equisetum arvense*), to 15,300 in the untreated cotton (Gossypium) fiber [14,15]. The hydroxyls linked to the hemiacetalic ring are arranged in an equatorial position that enables extremely stable conformation [16]. The substituents in adjacent rings are in a quasi-planar disposition that allows for the formation of a linear chain on which adjacent chains are aligned in a hydrogen-bond crystalline structure, with a hydrophilic character on the surface but hydrophobic in the interior, making cellulose insoluble in water [14,17,18].

Cellulose can be obtained from many sources, such as plants or microorganisms. Bacterial cellulose has attracted the attention of researchers in the last years. It consists of a gelatinous translucid pellicle grown on the surface of Acetobacter bacterial colonies. These microbial cellulose nanofibrils are about 280 nm in width and several micrometers in length [19]. The amorphous regions in this cellulose occupy almost 90% of its volume; however, as these regions contain up to 99% water, their contribution to final cellulose mass is minimal. BC’s overall crystallinity is high and is estimated to be about 70% [18,20]. BC’s water retention capacity is much better than cellulose from other sources, above 1000% [21]. To enable the oxygenation of tissues and fluid draining, the use of controlled production of variable size pores is necessary [22]. The abovementioned BC properties and their biocompatibility increased interest in using it for therapeutic purposes.

New studies on cellulose nanomaterials for tissue engineering have employed BC because it is a material with unique properties compared to biomaterials commonly used in tissue engineering scaffolds [12,21,23]. For instance, BC has been used in scaffolds applied in microsurgeries to promote burned tissue regeneration. The advancement of micro- and nanotechnologies enables developing tissue scaffolds with a gradient in material composition and properties that facilitate spatially controlled differentiation of cells and subsequent tissue development [23].

Nanofiber fabrication systems have been developed to mimic such fibrous structures of in vitro cell culture for the generation of polymer or composite fibers from natural or synthetic materials. These nanofibers possess a large surface area, which allows cell attachment [24,25,26,27]. Nanofibers’ physical and chemical properties can easily be tunable under appropriate conditions to facilitate cell growth and subsequent tissue development, thereby imparting gradient features into a nanofiber system, providing an exciting area of research [25,28]. Micro- and nanoscale techniques are versatile tools for developing such gradient biomaterials and can be utilized to design a new generation of engineered grafts for use in interface tissue engineering [25,29]. The membrane of BC has recently been shown to promote cellular adhesion, proliferation of skeletal muscle and mesenchymal stem cells, and angiogenesis during tissue regeneration [30,31,32]. These approaches can enable the modification of targeted structures via pharmaceutical molecules. For instance, BC and fluconazole in scaffolds have been used to promote stem cell growth during regeneration of burned tissues [33].

Emerging cell therapeutic strategies are promising procedures for promoting myocardial regeneration and repair. Different isolated or combined stem cells have been studied both in preclinical models and in clinical trials, including skeletal muscle cells (SMC), mesenchymal stem cells from adipose tissue, hematopoietic cells from the bone marrow, or umbilical cord blood (or Wharton Jelly) [34]. Various routes have been used to administer cells such as catheterization, and epicardial and intramyocardial injection. In addition, cell therapy has been suggested to be greatly improved if accompanied by a 3D scaffold, matrix modifiers, and adhesion molecules [35].

Carvalho et al. [34] have reported beneficial effects after cellular therapy by injecting cocultured bone marrow mesenchymal stem cells and skeletal muscle cells (BMSC) in Chagas myocardial disease and myocardial infarction (MI) models. They demonstrated improved functional and histopathologic outcomes caused by angiomuscular regeneration. These studies provided us with important information required for choosing and applying the cells used in this study. This study aimed to assess the potential improvement of infarcted heart function using autologous BMSC seeded on a BC patch. To our knowledge, this is the first study that uses BC membrane as a patch for cellular delivery in MI model.

## 2. Materials and Methods

### 2.1. Animals

The experimental animal protocol of this study was approved by the Pontifical Catholic University of Paraná Animal Use Committee, numbered 555 (CEUA-PUCPR). The rats were housed under standard conditions with food and water ad libitum on a 12-h day/night cycle (light on at 7 am). All animal experiments were performed at the Experimental Laboratory of the Institute of Biological and Health Sciences at the Pontifical Catholic University of Paraná. The facility was structured for animal housing as well as with animal experimental surgical room for the proceedings. All experiments approved were performed following the Animal Research: Reporting of In Vivo Experiments (ARRIVE) Guidelines [36].

### 2.2. Experimental Design

**Figure 1:**
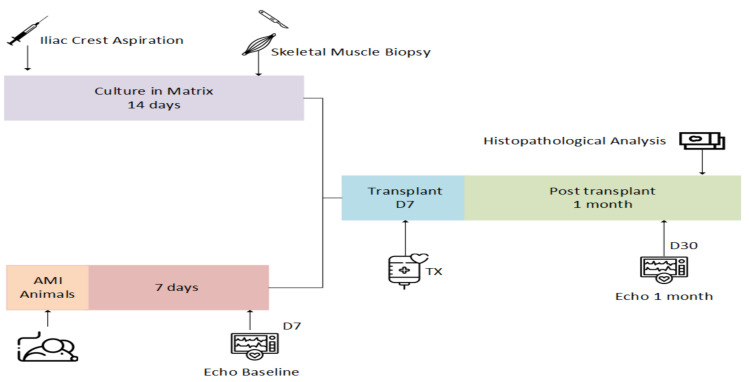
Experimental design: acute myocardial infarction (MI), distinct time point (D), echocardiography (Echo), and transplantation (TX).

The animals were obtained from the Pontifical Catholic University of Paraná. Forty-five male Wistar rats, *Rattus norvegicus* (weight, 250–300 g), were subjected to MI surgery, as described earlier [34]. Seven days after, they were analyzed by an echocardiography apparatus to assess baseline heart function. The animals that displayed ≤30% left ventricular ejection fraction (LVEF) were randomized into three groups: group I (*n* = 10), MI without treatment (control group); group II (*n* = 11), implantation of the BC matrix on the left ventricular surface; and group III (*n* = 11), implantation of the BC matrix seeded with cocultured cells on the left ventricular surface. Seven days after surgery, the second operation and implantation of the patch were performed. One month after MI, the hearts were analyzed by an echocardiography apparatus for a second time. The animals were then euthanized, and histopathological analysis was performed (Figure 1). Thirteen animals were discharged because they did not reveal MI criteria with ≤30% LVEF before randomization.

### 2.3. Acute Myocardial Infarction Model

The rats were anesthetized by intramuscular administration of ketamine (Dopalen^®^, Ceva Santé Animale, Paulínea, SP, Brazil) (50 mg/kg) and xylazine (Anasedan^®^, Ceva Santé Animale, Paulínea, SP, Brazil) (10 mg/kg) and were then subjected to mechanical ventilation. These procedures were followed by thoracotomy at the left, fifth intercostal space. To induce MI (D0), the left anterior descending (LAD) coronary artery was directly ligated just beyond the first diagonal branch. The rats were then maintained in cages and kept under controlled temperature and high oxygen pressure to facilitate postoperative recovery. Both normal respiratory activity and heart rate (350–450 beats/min) were carefully monitored before housing the animals under standard conditions until the next experimental step.

**Figure 2:**
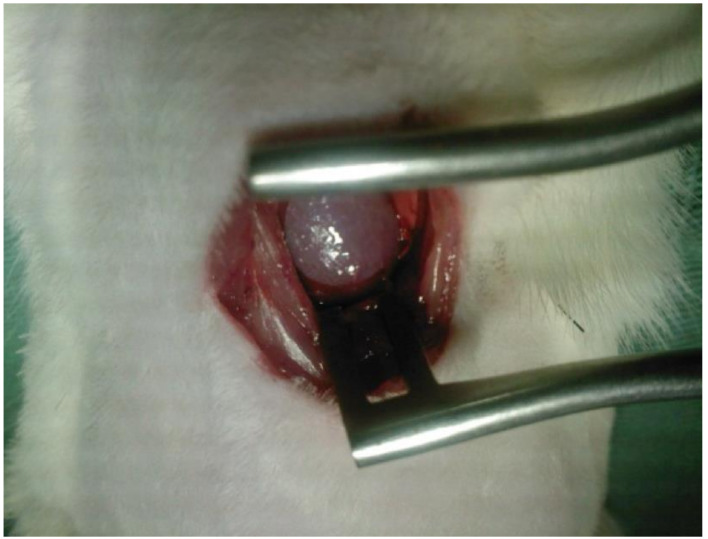
Placement of the cellulose patch on the left ventricle.

Seven days after surgery, the rats were subjected to another left thoracotomy (T2) for membrane implantation in the ventricular surface, with or without cocultured cells. Immediately after the LAD ligation, a bacterial membrane fragment was gently placed onto the left ventricle of group III animals, combined with cocultured cells in contact with the epicardial surface. The membrane fragment edges were ligated to the ventricle using a suture and placed without any artificial reinforcing effect. Finally, the sternum and skin incisions were sutured (Figure 2).

### 2.4. Euthanasia

All animals were euthanized with a lethal dose of pentobarbital sodium (thiopental) 200 to 250 mg/kg injected intraperitoneally.

### 2.5. Echocardiographic Analysis

The rats were first anesthetized (with 10 mg/kg xylazine and 50 mg/kg ketamine, intramuscular injection) and subjected to transthoracic Doppler echocardiographic studies using a Sonos 5500 (Agilent^®^, Santa Clara, CA, USA) echocardiographic model, equipped with a phased array 12–5 MHz probe, with a software specifically designed for studies in small animals. Local and overall left ventricular contractility was evaluated by assessing left ventricular ejection fraction (LVEF), left ventricular end-systolic volume (LVES), and left ventricular end-diastolic volume (LVED). The echocardiographic analysis was performed 7 days after MI (baseline, D7), 1 month after the MI, and after transplantation of the patch with or without cells (D30) [37,38].

### 2.6. Cell Isolation Procedures

Skeletal myoblasts were isolated after taking a biopsy from the lower limb’s skeletal muscle, as described earlier [39]. Bone marrow mesenchymal stem cells were isolated via bone marrow aspiration of the iliac crest [40]. After cell centrifugation, the isolation was performed using a density gradient, 1.077, Ficoll-Paque PLUS solution (Cytiva^®^, Piscataway, NJ, USA); then, mononuclear cell fractions were distributed in flasks, kept for 48 h, and washed with PBS (Gibcco Invitrogen^®^, Life Technologies, Inc, Rockville, MD, USA). Only the mesenchymal stem cells remained adherent, while cells of hematopoietic origin did not. For both cell types, the culture medium used was Dulbecco’s Modified Eagle Medium (DMEM) (Gibcco Invitrogen^®^ Life Technologies, Inc, Rockville, MD, USA) containing 15% fetal bovine serum (FBS) (Gibco Invitrogen^®^, Life Technologies, USA), 100 U/mL penicillin, and 100 μg/mL streptomycin (Gibcco Invitrogen^®^, Life Technologies, Inc, Rockville, MD, USA). The cultures were maintained in an incubator at 37 °C with 5% CO_2_.

The assays were performed in 25-cm^2^ polystyrene flasks (TPP^®^, Trasadingen, Switzerland). The BC (Membracel, Vuelo Pharma^®^, Almirante Tamandaré, PR, Brazil) and cells were seeded as described by Carvalho et al. [40]. The proportion of skeletal muscle cells to bone marrow mesenchymal stem cells was 2:1, approximately 5 × 10^5^/mL per 14 days. The coculture medium used was DMEM containing 15% FBS, 1% of antibiotics (100 U/mL penicillin and 100μg/mL streptomycin), and 10 ng/mL of insulin growth factor (IGF-I) (Gibcco BRL^®^, Life Technologies, Inc, Rockville, MD, USA). The medium was changed every 48 h, and the cultures were incubated at 37 °C with 5% CO_2_ [39].

### 2.7. MTT Assay

Cells were cocultured in the abovementioned media (1 × 10^4^ cm^2^) on the membrane surface and maintained in 6-well plates. The plates were incubated in standard cell culture conditions at 37 °C temperature with 5% CO_2_. Subsequently, 100 μL of 5 mg/mL 3-(4,5-dimethyl-2-thiazolyl)-2,5-diphenyl-2*H*-tetrazolium bromide (MTT) (final concentration 0.5 mg/mL) was added to the wells, and the cells were incubated at 37 °C with 5% CO_2_ for 1, 3, 7, and 10 days. The supernatants were utilized for analysis using a spectrophotometry reader (reference wavelength set to 595 and 630 nm) [40].

### 2.8. Cytometric Analysis

To verify the bone marrow mesenchymal stem cell origin and skeletal muscle cells, flow cytometric analysis was performed using the FACSCalibur system (BD Biosciences, San Jose, CA, USA). Immunophenotypic analyses for CD34, CD 45, CD105, CD 90, CD73, and Myo-D were performed with a commercially available kit (Stem Kit, Beckmann Coulter, Krefeld, Germany) as a single-platform method according to the International Society for Cellular Therapy (ISCT) [41]. This kit consisted of anti-CD45-FITC monoclonal antibody (Mab), anti-CD34-PE, CD 105-FITC (clone 266), CD 90-PE (clone OX-7), CD 73-PE, and CD Myo-D-FITC (clone Mab5.8A). The conjugated Mabs were provided in combinations ready to use.

### 2.9. Histopathological Analysis

The hearts were harvested and fixed in 10% neutral buffered formalin for 24 h. Sections were routinely stained with H&E and Gomory trichrome. To identify the cells, formalin-fixed and paraffin-embedded tissue sections were immunostained using the Vector^®^ M.O.M. Immuno-detection kit (Vector, Burlingame, CA, USA), and monoclonal antibodies specific for muscle alpha-actin (SR 1) and BrdU (BrdU in situ detection kit) (BD Biosciences, San Jose, CA, USA), according to the manufacture instructions. The primary antibody was applied for 1 h at room temperature. Angiogenesis was detected by immunoperoxidase staining for a vascular endothelial growth factor (VEGF) (Abcam, Cambridge, United Kingdom). The slides were then incubated with secondary biotin-labeled, affinity-isolated anti-rabbit and anti-mouse immunoglobulins (LSAB^®^ + Kit, Peroxidase; DAKO Corp, Carpinteria, CA, USA).

### 2.10. Scanning Electron Microscopy (SEM) Analysis

Cocultured cells (1 × 10^4^ cm^2^) were grown on a BC patch for 14 days. The patch was rinsed three times with PBS; fixed in 2% paraformaldehyde, 2.5% glutaraldehyde in a 0.1 M phosphate buffer for 15 min; and rinsed in distilled water. Dehydration was performed in a series of ethanol concentrations (50%, 70%, 90%, and twice in 100%). The dehydrated specimens were kept overnight in a vacuum oven at 25 °C, after which they were support-coated with gold and examined with A JEOL 6360LV SEM, Japan, Tokyo, Japan, at a 30 kV accelerating voltage. The experiment was repeated four times, and respective photographs were taken (*n* = 5).

### 2.11. Statistical Analysis

All numerical data are shown as mean values *p*/95% confidence limits. Statistical significance was assessed using a one-way analysis of variance (ANOVA), and the minimum significant difference between means of each group was calculated using the *T*-test method. For a comparison of 2 groups, a 2-tailed unpaired Student *t*-test was used; the condition of normality was assessed using the Kolmogorov–Smirnov test. *p*-values ≤ 0.05 were considered statistically significant.

## 3. Results

### 3.1. Cell Adhesion and Proliferation

**Figure 3:**
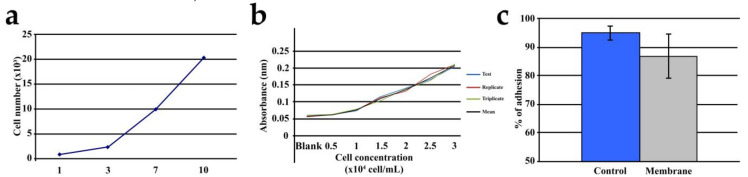
(**a**). Cell proliferation analysis during 10 days of cell culture on the cellulose membrane. (**b**). MTT (3-(4,5-dimethyl-2-thiazolyl)-2,5-diphenyl-2*H*-tetrazolium bromide) analysis showing the standard curve for cell proliferation. (**c**). Cell adhesion rate, represented by mean ± SD. The control group mean was 95.17% ± 2.44, whereas the membrane group mean was 87.07% ± 7.77. Control group: polystyrene flask culture. Membrane: cellulose membrane.

Regular monitoring of the cocultured cells confirmed that they adhered very well to the BC membranes. We analyzed the proliferation of cocultured cells using an MTT assay and found that the BC membranes could support coculture growth and adhesion (Figure 3a–c).

**Figure 4:**
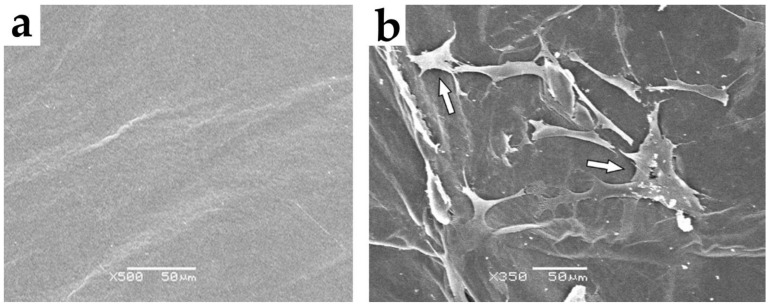
(**a**). SEM analysis showing bacterial cellulose (BC) membrane without cells. Images 500×, scale bar = 50 μm. (**b**). SEM analysis of the BC membrane containing cocultured skeletal muscle cells and bone marrow mesenchymal stem cells (for 14 days). Arrows indicate cell adhesion. Images 350×, scale bar = 50 μm.

The cocultured cells on the BC membranes showed exponential growth over 14 days. The changes in the membrane, with or without cells, were not due to toxicity as confirmed by SEM analyses, which showed superior cell growth and spread throughout the cocultured BC membranes (Figure 4a,b).

### 3.2. Echocardiographic Findings

**Figure 5:**
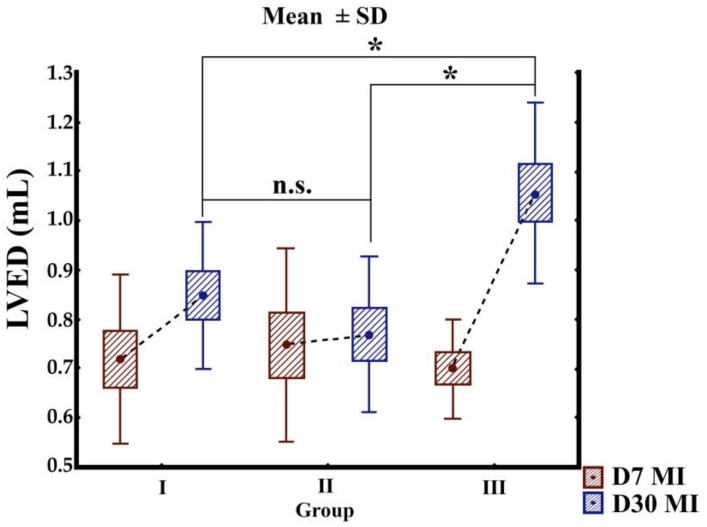
Graphs show echocardiographic measurements of left ventricular end-diastolic (LVED). Mean values were calculated for each group seven days (D7) after and 30 (D30) days after MI. Group I: MI without treatment as a control group; group II: implantation of the BC matrix on the left ventricular surface; and group III: Implantation of the BC matrix seeded with cocultured cells on the left ventricular surface. Standard Deviation: SD, Ns: not significant, asterisk: significant.

**Figure 6:**
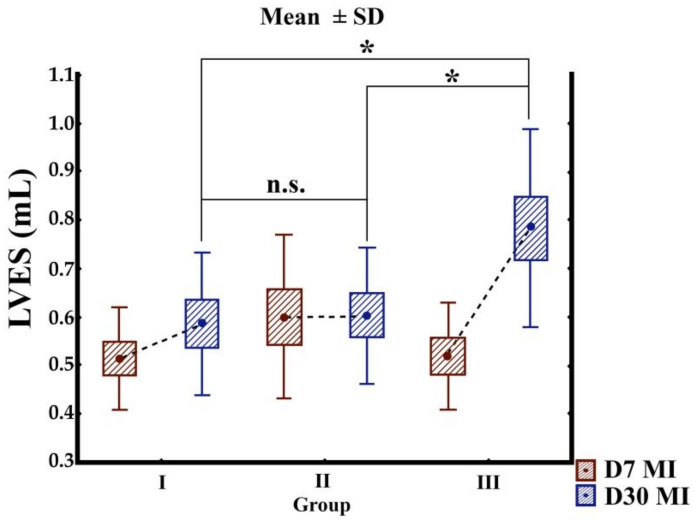
Graphs show echocardiographic measurements of left ventricular end-systolic (LVES). Mean values were calculated for each group seven days (D7) after and 30 (D30) days after MI. Group I: MI without treatment as a control group; group II: implantation of the BC matrix on the left ventricular surface; and group III: Implantation of the BC matrix seeded with cocultured cells on left ventricular surface. ns: not significant. Asterisk: significant.

Three groups were used in this study, for a total of 32 animals: group I (*n* = 10), MI without treatment (control group); group II (*n* = 11), implantation of the BC matrix on the left ventricular surface; and group III (*n* = 11), implantation of the BC matrix seeded with cocultured cells on the left ventricular surface. Mortality of the rats of this study after MI was one in group I and two in group II. Left ventricular (LV) function was analyzed seven days after MI (baseline) (D7) and 30 days after infarction (D30). At baseline, the mean ejection function, LVES, and LVED were similar in the three groups: *p* = 0.863, *p* = 0.302, and *p* = 0.798, respectively. Over the course of 30 days, the membrane implantation decreased the LVED dimension compared with the baseline value (Table 1 and Figure 4). Additionally, a significant attenuation of LV dilatation was achieved when the membrane was implanted without cells (group II) compared with the two other groups. There was no detectable dilation of the left ventricle in the untreated control and the membrane groups, as revealed by decreased LVED and LVES (Figure 5 and Figure 6). In contrast to the cellulose patch group, the control samples revealed remarkable remodeling of the myocardium. The cellulose patch group did not reveal any improvement in left ventricular ejection fraction compared with the patch with cocultured cells and with the control (Table 1, and Figure 5 and Figure 6).

### 3.3. Histopathological Findings

Assessment of the heart chambers, externally and internally, showed ischemic lesions in the left ventricle’s anterior wall and the interventricular septum, with no apparent differences between samples, confirming the in vivo morphologic and functional data.

**Figure 7:**
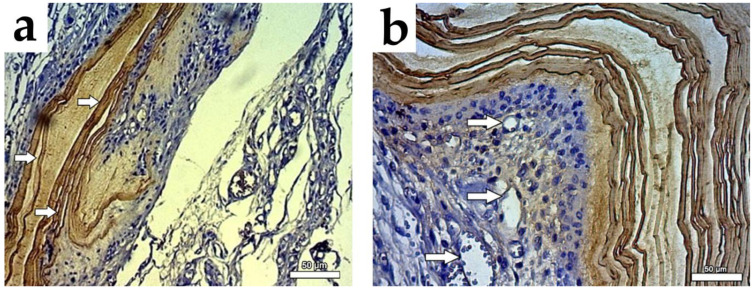
(**a**). Immunostaining of the post-transplantation cardiac scar tissue. Sections were stained with anti-BrdU antibodies after 30 days of infarction without cells (group II). The arrows point to the cellulose membrane implanted as a patch; 400× (optical light microscopy). (**b**). Cocultured cells grafted in the post-transplantation cardiac scar tissue. Sections were stained with anti-BrdU antibodies (dark) after 30 days of infarction (group III). The arrows indicate engrafted cocultured cells and vessels; 400× (optical light microscopy).

To characterize the cells used in our experimental model, histopathological analysis was performed and indicated that the isolated cocultured cells adhered to the BC membrane and acquired a skeletal morphology after 14 days in culture (Figure 3a,b). We also detected remarkable proliferation of the cocultured cells (BrdU labeling) accompanied by evident angiogenesis (VEGF labeling) (Figure 7a,b). We continued our cellular assessment via SEM analysis, which indicated that cocultured cells adhered well and grew intensively in the cellulose membrane (Figure 4a,b).

## 4. Discussion

The present results show that using a cellulose patch without cells can protect the myocardium against deleterious effects and pathological remodeling of the ischemic heart. Our findings demonstrated that cellulose patches combined with cells result in beneficial effects not provided by cell therapy.

To mimic fibrous structures of an in vitro cell culture, nanofiber fabrication systems have been developed to generate a polymer or composite fibers from natural or synthetic materials. These nanofibers possess a large surface area, which is favorable for cell attachment [16]. Nanofibers’ physical and chemical properties can easily be tunable under appropriate conditions to facilitate cell growth and subsequent tissue development, thereby imparting gradient features into a nanofiber system and thus offering an exciting area of research [20,28,32]. Micro- and nanoscale techniques are versatile tools for developing such gradient biomaterials and could be utilized to design a new generation of engineered grafts for use in interface tissue engineering [12,25].

The findings of this study could be developed further by investigating the incorporation of biomolecules as growth factor TGF-β1, which is known to promote cell differentiation and proliferation. Additionally, the use of bioinformatics databases have uncovered the expression and effect of genes generally implicated in tissue regeneration such as TGF β1, MMP2, MMP9, CTNNB1, Wnt4, hsa-miR-29b-3p, and hsa-miR-29c-3p [42]. Gene expression studies have been developed to ensure successful tissue regeneration, such as using BC to release BMP-2 and to promote optimal tissue formation. Interestingly, the BC + BMP-2 combination enhanced bone regeneration and appeared to be a promising clinical approach [43]. These tools may help preserve the native phenotype, which is considered a complex challenge in the field.

On the other hand, studies have suggested benefits of cocultured cell transplantation in promoting cardiac tissue regeneration and restoring the infarcted heart [25,28]. Previous preclinical studies and clinical trials have used different cell types and biomaterials to test the cellular therapeutic effect on cardiac repair and reported promising results [44,45,46,47,48,49,50]. Several types of cells have been proposed for cardiomyoplasty [33,45,47,50]. Using an autologous model of cocultured skeletal muscle cells and bone marrow mesenchymal stem cells is based on cell characteristics. While skeletal muscle cells are known to be resistant to ischemia, mesenchymal stem cells were shown to be considerably angiogenic. Thus, combining these cells would enable better angiomuscular myocardial regeneration [51,52,53]. Schussler et al. [25] have shown that transplantation of stem cells combined with three-dimensional (3D) collagen scaffolds into ischemic rat hearts can prevent and reverse heart failure progression. Other investigators have used echocardiography and histology and reported that the collagen matrix in MI models did not improve pathological post-ischemic remodeling, ejection fraction (EF), and LV wall thickness. However, the latter authors found that using a combination of matrix and cells could prevent ventricular wall thinning [54]. Recently, BC has been demonstrated to enhance the adhesion and proliferation of skeletal muscle and mesenchymal cells [31]. The cellulose patch likely supports cells known to produce paracrine effects in situ or allows for the mobilization of autologous resident stem cells to the site of injury, as shown in other systems [54,55,56,57]. In conclusion, the present study suggests that using cellulose as a patch is effective for cell delivery into the myocardium, preventing deleterious remodeling of the ischemic heart. Thus, the cellulose patch is a biomaterial with significant potential in repairing heart damage. Further studies will be necessary to evaluate these beneficial effects in larger animals to facilitate clinical translation.

## Figures and Tables

**Table 1:** Echocardiographic results in the three groups at two time-points.

Variable	Group	D7	D30	*p*-Value (D7 vs. D30)
LVEF (%)	I	28.21 ± 5.07	32.72 ± 8.62	0.316
	II	22.79 ± 5.80	26.74 ± 4.92	0.065
	III	27.37 ± 8.40	27.92 ± 9.77	0.865
LVES (mL)	I	0.509 ± 0.106	0.582 ± 0.149	0.241
	II	0.596 ± 0.171	0.598 ± 0.141	0.982
	III	0.514 ± 0.112	0.780 ± 0.206	0.001 *
LVED(mL)	I	0.718 ± 0.170	0.848 ± 0.149	0.326
	II	0.747 ± 0.196	0.768 ± 0.158	0.043
	III	0.698 ± 0.102	1.056 ± 0.183	0.004 *

Note: Distinct time point (D); left ventricular fraction ejection (LVEF); left ventricular end-systolic (LVES); left ventricular end-diastolic (LVED). Data are shown as mean ± SD. *P* < 0.05 was considered statistically significant. Group I: MI without treatment as a control group; group II: implantation of the BC matrix on the left ventricular surface; and group III: implantation of the BC matrix seeded with cocultured cells on the left ventricular surface. *: significant.

## Data Availability

Not applicable.

## References

[1] Cunningham J.W., Vaduganathan M., Claggett B.L., John J.E., Desai A.S., Lewis E.F., Zile M.R., Carson P., Jhund P.S., Kober L. (2020). Myocardial Infarction in Heart Failure With Preserved Ejection Fraction: Pooled Analysis of 3 Clinical Trials. JACC Hear. Fail..

[2] Mahoney D.W., Jacobsen S.J., Rodeheffer R.J., Burnett J.C., Redfield M.M., Bailey K.R. (2003). Burden of Systolic and Diastolic Ventricular Dysfunction in the Community. JAMA.

[3] Pfeffer M.A., Braunwald E. (1990). Ventricular remodeling after myocardial infarction: Experimental observations and clinical implications. Circulation.

[4] Abdelwahid E., Kalvelyte A., Stulpinas A., De Carvalho K.A.T., Guarita-Souza L.C., Foldes G. (2016). Stem cell death and survival in heart regeneration and repair. Apoptosis.

[5] Granero-Molto F., Weis J.A., Longobardi L., Spagnoli A. (2008). Role of mesenchymal stem cells in regenerative medicine: Application to bone and cartilage repair. Expert Opin. Biol. Ther..

[6] Melchels F.P.W., Tonnarelli B., Olivares A.L., Martin I., Lacroix D., Feijen J., Wendt D.J., Grijpma D.W. (2011). The influence of the scaffold design on the distribution of adhering cells after perfusion cell seeding. Biomaterials.

[7] Su G., Zhao Y., Wei J., Han J., Chen L., Xiao Z., Chen B., Dai J. (2013). The effect of forced growth of cells into 3D spheres using low attachment surfaces on the acquisition of stemness properties. Biomaterials.

[8] Chen R. (2015). A paradigm shift in biomass technology from complete to partial cellulose hydrolysis: Lessons learned from nature. Bioengineered.

[9] Ross P., Mayer R., Benziman A.N.D.M. (1991). Cellulose biosynthesis and function in bacteria positive control. Microbiol. Rev..

[10] Svensson A., Nicklasson E., Harrah T., Panilaitis B., Kaplan D.L., Brittberg M., Gatenholm P. (2005). Bacterial cellulose as a potential scaffold for tissue engineering of cartilage. Biomaterials.

[11] Tabata Y. (2009). Biomaterial technology for tissue engineering applications. J. R. Soc. Interface.

[12] Frazier T., Alarcon A., Wu X., Mohiuddin O.A., Motherwell J.M., Carlsson A.H., Christy R.J., Edwards J.V., Mackin R.T., Prevost N. (2020). Clinical translational potential in skin wound regeneration for adipose-derived, blood-derived, and cellulose materials: Cells, exosomes, and hydrogels. Biomolecules.

[13] Feil G., Horres R., Schulte J., Mack A.F., Petzoldt S., Arnold C., Meng C., Jost L., Boxleitner J., Kiessling-Wolf N. (2017). Bacterial cellulose shifts transcriptome and proteome of cultured endothelial cells towards native differentiation. Mol. Cell. Proteomics.

[14] Fengel D., Wegner G. (1989). Wood Chemistry, Ultrastructure, Reactions.

[15] Shafizadeh F. (1983). Wood chemistry: Fundamentals and applications. Carbohydr. Res..

[16] Solomons T.W.G., Fryhle C.B., Snyder S.A. (1996). Carbohydrates. Organic Chemistry.

[17] Klemm D., Heublein B., Fink H.P., Bohn A. (2005). Cellulose: Fascinating biopolymer and sustainable raw material. Angew. Chem. Int. Ed..

[18] Zugenmaier P. (2001). Conformation and packing of various crystalline cellulose fibers. Prog. Polym. Sci..

[19] George J., Ramana K.V., Bawa A.S. (2011). Siddaramaiah Bacterial cellulose nanocrystals exhibiting high thermal stability and their polymer nanocomposites. Int. J. Biol. Macromol..

[20] Koizumi S., Yue Z., Tomita Y., Kondo T., Iwase H., Yamaguchi D., Hashimoto T. (2008). Bacterium organizes hierarchical amorphous structure in microbial cellulose. Eur. Phys. J. E.

[21] Klemm D., Schumann D., Udhardt U., Marsch S. (2001). Bacterial synthesized cellulose - Artificial blood vessels for microsurgery. Prog. Polym. Sci..

[22] Fontana J.D., De Souza A.M., Fontana C.K., Torriani I.L., Moreschi J.C., Gallotti B.J., De Souza S.J., Narcisco G.P., Bichara J.A., Farah L.F.X. (1990). Acetobacter cellulose pellicle as a temporary skin substitute. Appl. Biochem. Biotechnol..

[23] Dugan J.M., Gough J.E., Eichhorn S.J. (2013). Bacterial cellulose scaffolds and cellulose nanowhiskers for tissue engineering. Nanomedicine.

[24] Yang F., Murugan R., Wang S., Ramakrishna S. (2005). Electrospinning of nano/micro scale poly(l-lactic acid) aligned fibers and their potential in neural tissue engineering. Biomaterials.

[25] Seidi A., Ramalingam M., Elloumi-Hannachi I., Ostrovidov S., Khademhosseini A. (2011). Gradient biomaterials for soft-to-hard interface tissue engineering. Acta Biomater..

[26] Atyabi S.M., Sharifi F., Irani S., Zandi M., Mivehchi H., Nagheh Z. (2016). Cell Attachment and Viability Study of PCL Nano-fiber Modified by Cold Atmospheric Plasma. Cell Biochem. Biophys..

[27] Ziaei Amiri F., Pashandi Z., Lotfibakhshaiesh N., Mirzaie Parsa M.J., Ghanbari H., Faridi-Majidi R. (2020). Cell attachment effects of collagen nanoparticles on crosslinked electrospun nanofibers. Int. J. Artif. Organs.

[28] Rajwade J.M., Paknikar K.M., Kumbhar J.V. (2015). Applications of bacterial cellulose and its composites in biomedicine. Appl. Microbiol. Biotechnol..

[29] Liu Z., Cui A., Li J., Gu C. (2019). Folding 2D Structures into 3D Configurations at the Micro/Nanoscale: Principles, Techniques, and Applications. Adv. Mater..

[30] Ortega Z., Alemán M.E., Donate R. (2018). Nanofibers and Microfibers for Osteochondral Tissue Engineering. Adv. Exp. Med. Biol..

[31] Baggio Simeoni R., Cesar Francisco J., Cunha R., André Cardoso M., Athayde T Carvalho K., Guarita–Souza L.C. (2016). Co-cultivated cells integration into bacterial cellulose scaffold as a new device for tissue regeneration. Front. Nanosci. Nanotechnol..

[32] Wang B., Lv X., Chen S., Li Z., Yao J., Peng X., Feng C., Xu Y., Wang H. (2018). Use of heparinized bacterial cellulose based scaffold for improving angiogenesis in tissue regeneration. Carbohydr. Polym..

[33] Souza C.M.C.O., Mesquita L.A.F., Souza D., Irioda A.C., Francisco J.C., Souza C.F., Guarita-Souza L.C., Sierakowski M.R., Carvalho K.A.T. (2014). Regeneration of skin tissue promoted by mesenchymal stem cells seeded in nanostructured membrane. Transplant. Proc..

[34] Carvalho K.A.T., Guarita-Souza L.C., Hansen P., Rebelatto C.L.K., Senegaglia A.C., Miyague N., Olandoski M., Francisco J.C., Furuta M., Gremski W. (2006). Cell Transplantation After The Coculture of Skeletal Myoblasts and Mesenchymal Stem Cells in the Regeneration of the Myocardium Scar: An Experimental Study in Rats. Transplant. Proc..

[35] Schussler O., Chachques J.C., Mesana T.G., Suuronen E.J., Lecarpentier Y., Ruel M. (2010). 3-Dimensional Structures To Enhance Cell Therapy and Engineer Contractile Tissue. Asian Cardiovasc. Thorac. Ann..

[36] Kilkenny C., Browne W.J., Cuthill I.C., Emerson M., Altman D.G. (2013). Improving bioscience research reporting: The arrive guidelines for reporting animal research. Animals.

[37] Anversa P., Capasso J.M., Puntillo E., Sonnenblick E.H., Olivetti G. (1989). Morphometric Analysis of the Infarcted Heart. Pathol. Res. Pract..

[38] Rudski L.G., Lai W.W., Afilalo J., Hua L., Handschumacher M.D., Chandrasekaran K., Solomon S.D., Louie E.K., Schiller N.B. (2010). Guidelines for the Echocardiographic Assessment of the Right Heart in Adults: A Report from the American Society of Echocardiography. Endorsed by the European Association of Echocardiography, a registered branch of the European Society of Cardiology, and the Canadian Society of Echocardiography. J. Am. Soc. Echocardiogr..

[39] Delaporte C., Fardeau M. (1984). [The effect of serum from a patient with myeloma and diffuse muscular hypertrophy on the growth of human muscle cells in culture]. C. R. Acad. Sci. III.

[40] Carvalho K.A.T., Guarita-Souza L.C., Rebelatto C.L.K., Senegaglia A.C., Hansen P., Mendonça J.G.R., Cury C.C., Francisco J.C., Brofman P.R.S. (2004). Could the coculture of skeletal myoblasts and mesenchymal stem cells be a solution for postinfarction myocardial scar?. Transplant. Proc..

[41] Dominici M., Le Blanc K., Mueller I., Slaper-Cortenbach I., Marini F.C., Krause D.S., Deans R.J., Keating A., Prockop D.J., Horwitz E.M. (2006). Minimal criteria for defining multipotent mesenchymal stromal cells. The International Society for Cellular Therapy position statement. Cytotherapy.

[42] Moniri M., Moghaddam A.B., Azizi S., Rahim R.A., Zuhainis S.W., Navaderi M., Mohamad R. (2018). In vitro molecular study of wound healing using biosynthesized bacteria nanocellulose/ silver nanocomposite assisted by bioinformatics databases. Int. J. Nanomedicine.

[43] Koike T., Sha J., Bai Y., Matsuda Y., Hideshima K., Yamada T., Kanno T. (2019). Efficacy of bacterial cellulose as a carrier of BMP-2 for bone regeneration in a rabbit frontal sinus model. Materials (Basel).

[44] Huang Q., Chen M., Liang S., Acha V., Liu D., Yuan F., Hawks C.L., Hornsby P.J. (2007). Improving cell therapy-experiments using transplanted telomerase-immortalized cells in immunodeficient mice. Mech. Ageing Dev..

[45] Donndorf P., Kundt G., Kaminski A., Yerebakan C., Liebold A., Steinhoff G., Glass A. (2011). Intramyocardial bone marrow stem cell transplantation during coronary artery bypass surgery: A meta-analysis. J. Thorac. Cardiovasc. Surg..

[46] Zimmermann W.H., Melnychenko I., Wasmeier G., Didié M., Naito H., Nixdorff U., Hess A., Budinsky L., Brune K., Michaelis B. (2006). Engineered heart tissue grafts improve systolic and diastolic function in infarcted rat hearts. Nat. Med..

[47] Zimmermann W., Eschenhagen T. (2003). Cardiac Tissue Engineering for Replacement Therapy. Heart Fail. Rev..

[48] Langer R. (2000). Tissue Engineering. Mol. Ther..

[49] Francisco J.C., Correa Cunha R., Cardoso M.A., Baggio Simeoni R., Mogharbel B.F., Picharski G.L., Silva Moreira Dziedzic D., Guarita-Souza L.C., Carvalho K.A.T. (2016). Decellularized Amniotic Membrane Scaffold as a Pericardial Substitute: An In Vivo Study. Transplant. Proc..

[50] Abdelwahid E., Siminiak T., Cesar Guarita-Souza L., Athayde Teixeira de Carvalho K., Gallo P., Shim W., Condorelli G. (2011). Stem Cell Therapy in Heart Diseases: A Review of Selected New Perspectives, Practical Considerations and Clinical Applications. Curr. Cardiol. Rev..

[51] Menasché P., Hagège A., Scorsin M., Pouzet B., Desnos M., Duboc D., Schwartz K., Vilquin J.T., Marolleau J.P. (2001). [Autologous skeletal myoblast transplantation for cardiac insufficiency. First clinical case]. Arch. Mal. Coeur Vaiss..

[52] Siu C.W., Liao S.Y., Liu Y., Lian Q., Tse H.F. (2010). Stem cells for myocardial repair. Thromb. Haemost..

[53] Tamaki T., Uchiyama Y., Okada Y., Tono K., Masuda M., Nitta M., Hoshi A., Akatsuka A. (2010). Clonal differentiation of skeletal muscle-derived CD34-/45 - stem cells into cardiomyocytes in vivo. Stem Cells Dev..

[54] Hagège A.A., Marolleau J.P., Vilquin J.T., Alhéritière A., Peyrard S., Duboc D., Abergel E., Messas E., Mousseaux E., Schwartz K. (2006). Skeletal myoblast transplantation in ischemic heart failure: Long-term follow-up of the first phase I cohort of patients. Circulation.

[55] Cortes-Morichetti M., Frati G., Schussler O., Van Huyen J.P.D., Lauret E., Genovese J.A., Carpentier A.F., Chachques J.C. (2007). Association between a cell-seeded collagen matrix and cellular cardiomyoplasty for myocardial support and regeneration. Tissue Eng..

[56] Soler-Botija C., Bagó J.R., Llucià-Valldeperas A., Vallés-Lluch A., Castells-Sala C., Martínez-Ramos C., Fernández-Muiños T., Chachques J.C., Pradas M.M.P., Semino C.E. (2014). Engineered 3D bioimplants using elastomeric scaffold, self-assembling peptide hydrogel, and adipose tissue-derived progenitor cells for cardiac regeneration. Am. J. Transl. Res..

[57] Zhang R., Mägel L., Jonigk D., Länger F., Lippmann T., Zardo P., Pölzing F. (2017). Biosynthetic Nanostructured Cellulose Patch for Chest Wall Reconstruction: Five-Month Follow-up in a Porcine Model. J. Investig. Surg..

